# Saturation association between serum 25-hydroxyvitamin D levels and mortality in elderly people with hyperlipidemia: a population-based study from the NHANES (2001-2016)

**DOI:** 10.3389/fendo.2024.1382419

**Published:** 2024-10-02

**Authors:** Guang-hui Pan, Jun-qing Zhang, Yi-yan Sun, Yue-hui Shi, Fa-rong Zhang

**Affiliations:** ^1^ Department of Nephrology, Affiliated Hospital of Shandong University of Traditional Chinese Medicine, Jinan, China; ^2^ College of First Clinical Medicine, Shandong University of Traditional Chinese Medicine, Jinan, China

**Keywords:** 25(OH)D serum levels, hyperlipidemia, the elderly, mortality, NHANES

## Abstract

**Background:**

25-hydroxyvitamin D is the body’s main storage form of vitamin D and is internationally recognized as the best indicator of vitamin D status in the human body. There is a scarcity of research investigating the interrelationship between serum 25-hydroxyvitamin D (25(OH)D) levels and mortality among elderly individuals with hyperlipidemia. To address this knowledge gap, we examined the association between serum 25(OH)D levels and mortality in an older hyperlipidemic population from NHANES, while controlling for other influential factors. The study sought to elucidate the correlation between serum 25(OH)D levels and mortality about all-cause mortality, cardiovascular disease (CVD), malignant neoplasms, and mortality from other causes.

**Methods:**

The data from NHANES 2001-2016, including 9,271 participants were analyzed to examine the association between serum 25(OH)D levels and mortality. The interrelationship was illustrated using Kaplan-Meier curves and restricted cubic splines, while the Cox proportional hazards model was utilized to estimate the multifactor adjusted hazard ratio (HR).

**Results:**

This study included 9,271 participants (43.28% male) with an average age of 69.58 years, and the average duration of participant follow-up was 88.37 months. Kaplan-Meier curves demonstrated that lower serum 25(OH)D levels were associated with increased risks of all-cause mortality, cardiovascular mortality, malignant neoplasm mortality, and mortality from other causes. This negative association was further confirmed by the Cox proportional hazards models. Additionally, restricted cubic splines not only revealed this negative association but also highlighted the saturated serum 25(OH)D levels. Moreover, subgroup analyses indicated that the inverse correlation between serum 25(OH)D levels and all-cause mortality was more pronounced in the non-obese and smoking population. And the inverse correlation with mortality from other causes was even stronger in the non-obese population.

**Conclusions:**

In the elderly population with hyperlipidemia, 25(OH)D serum levels were negatively correlated with both cause-specific mortality and all-cause mortality. Moreover, there was a threshold effect in this negative association.

## 1 Introduction

Hyperlipidemia is a metabolic disorder characterized by dysregulated lipid metabolism. It can result in elevated cholesterol or triglycerides in the bloodstream, which are usually associated with reduced levels of high-density lipoprotein (1). There are numerous studies demonstrated that elevated serum cholesterol, increased low-density lipoprotein (LDL) or triglyceride levels, and reduced serum high-density lipoprotein (HDL) levels are strongly associated with the increased risk of CVD in patients (2). In recent studies, it has been demonstrated that hyperlipidemia is also significantly associated with heightened mortality risk across multiple conditions, including diabetes, malignancies, systemic autoimmune diseases, and renal diseases (3–6). Currently, hyperlipidemia continues to present significant challenges to the global population, with a considerable proportion of patients receiving lipid-lowering treatment failing to achieve optimal control (7). Elderly individuals are a high-risk group for developing hyperlipidemia, which further amplifies their susceptibility to mortality from diverse pathological conditions (1).

Vitamin D is one of the most essential micronutrients of humans. 25(OH)D represents the primary form of vitamin D synthesized by the human body and serves as a key marker to evaluate the absorption and metabolism of vitamin D (8). However, its optimal level remains controversial. The current regulations consider that 25(OH)D laboratory analyses should be monitored for performance through an external quality assessment scheme that provides target reference values for standardized measurement procedures (9). Notably, serum levels of 25(OH)D are closely associated with various health conditions and prognoses. Extensive evidence suggests that lower levels of 25(OH)D are linked to an increased risk of developing conditions such as osteoporosis, chronic kidney diseases, autoimmune disorders, nervous system diseases, and specific types of cancers (10–13). Moreover, numerous research studies have consistently demonstrated a significant correlation between reduced 25(OH)D levels and elevated mortality risk. Similarly, serum levels of 25(OH)D exert an impact on the occurrence and progression of CVD and diabetes. Adequate levels of 25(OH)D have been shown to protect the cardiovascular system and help prevent illnesses such as hypertension, coronary artery disease, and stroke (14). Conversely, insufficient levels of 25(OH)D can contribute to insulin resistance and the development of diabetes, given its vital role in insulin secretion, the maturation of insulin-secreting cells, and overall insulin function. In summary, 25(OH)D serum levels have a significant impact on the overall health of populations.

In conclusion, we hypothesized that there might be a potential association between serum levels of 25(OH)D and mortality in the elderly population with hyperlipidemia. Therefore, this study aimed to investigate this potential association, thus contributing to improving the health status of older adults affected by hyperlipidemia.

## 2 Method

### 2.1 Study design and participants

NHANES (https://www.cdc.gov/nchs/nhanes/index.htm), conducted by the Centers for Disease Control and Prevention (CDC), is a survey research program based on the population and cross-sectional, which was used to evaluate the nutritional conditions and the overall health of the entire population in American through a complex survey method. The database includes a wide range of data, including demographic, dietary, examination, laboratory, and questionnaire information. The study procedures were authorized by the Ethical Review Committee of the National Health Statistical Research Center and all individuals agreed to join the study. Data collection was carried out over eight periods between 2001 and 2016. Additionally, the NHANES Public-Use Linked Mortality Files were linked with the NHANES database from 2001 to 2016, enabling researchers to identify participant deaths up until December 31, 2019.

**Figure 1:**
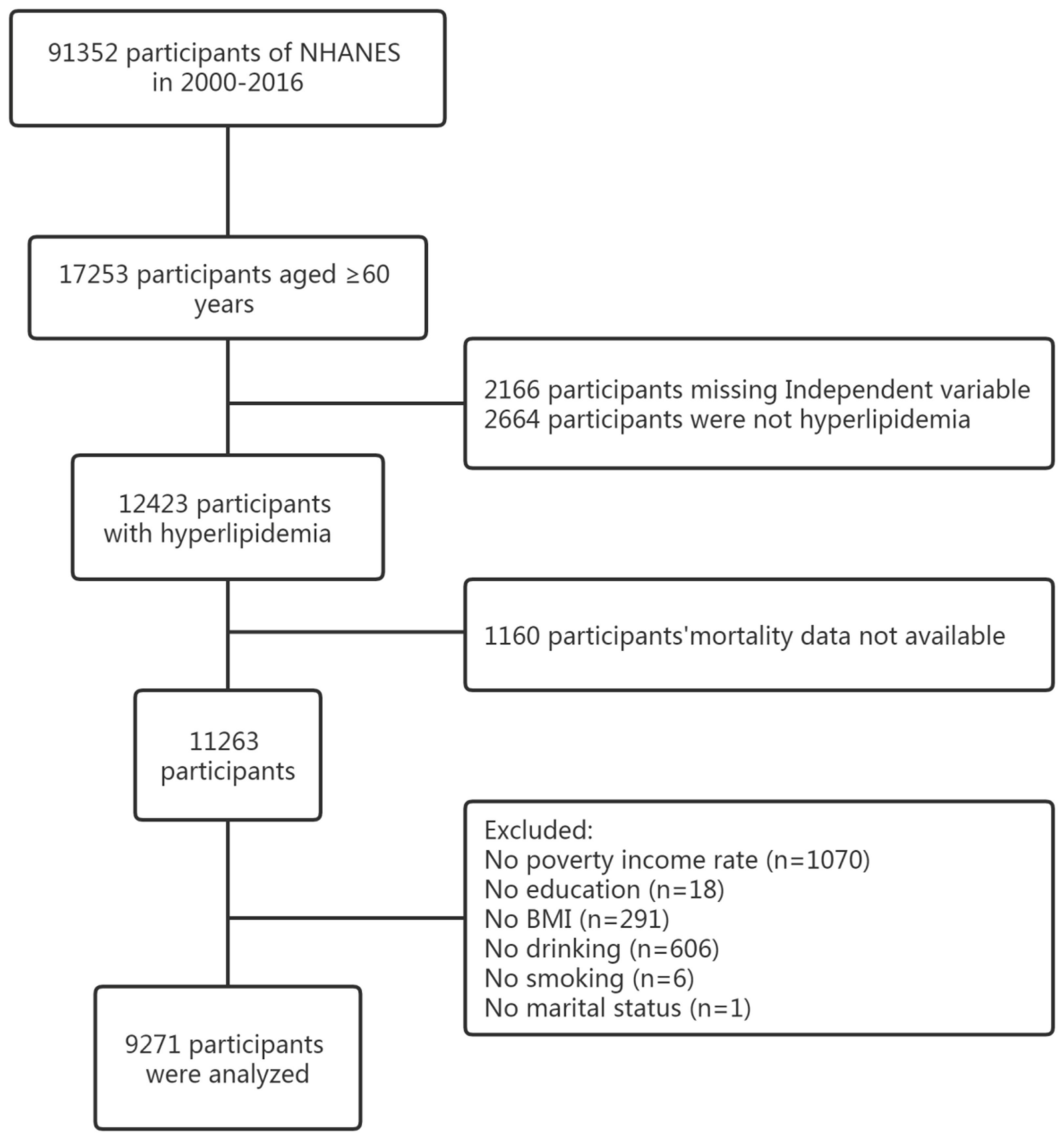
Flow chart of this study design from NHANES. NHANES, National Health and Nutrition Examination Survey.

In this cohort study, a total of 91,352 subjects were initially included. After including participants aged 60 years or older, we excluded participants with missing 25(OH)D information (n=2,166), participants with undiagnosed hyperlipidemia (n=2,664), and participants with missing mortality data (n=1,160). Subsequently, we further excluded participants with missing information on covariates, including those with missing information on poverty income rate (PIR), education information, body mass index (BMI), and information on drinking and smoking. A final analysis cohort of 9271 individuals was obtained (shown in **Figure 1**).

### 2.2 Determination of 25(OH)D serum levels

In the NHANES 2001-2006 study, 25(OH)D serum levels were measured by the CDC using radioimmunoassay (DiaSorin Assay Kit). Serum samples from 2007 to 2016 were analyzed using a standardized liquid chromatography-tandem mass spectrometry (LC-MS/MS) to estimate the 25(OH)D serum levels. The 25(OH)D serum levels of participants who took part in NHANES 2001-2006 were transformed into equivalent values derived from the standardized LC-MS/MS technique using regression. This transformation was performed to facilitate the utilization and evaluation of 25(OH)D serum levels by researchers. The unit of measurement for 25(OH)D serum levels is nmol/L.

### 2.3 Covariates

The study incorporated data from the NHANES database, which encompasses details on demographics, examination information, laboratory information, and questionnaire information. The following categories composed the demographic data: age, gender, ethnicity (Mexican American, Other Hispanic, Non-Hispanic Black, Non-Hispanic White, or Other Race), level of education (less than high school, high school or equivalent, and college or above), the status of marital (never married, married, and separated), and PIR. Age and PIR were treated as continuous variables, while race, education, and marital status were considered categorical variables. Examination information included diastolic blood pressure (DBP), systolic blood pressure (SBP), weight, and height information. BMI was calculated by dividing the participant’s weight in kilograms by the square of their height in meters. Laboratory information contained measurements for fasting plasma glucose, hemoglobin A1c (HbA1c), total cholesterol, triglycerides, HDL, and LDL. The questionnaire section contained information about drug use, alcohol consumption, and smoking habits.

In this study, hypertension was identified as a self-reported high blood pressure diagnosis, currently receiving treatment for hypertension, with SBP (average) ≥140 mmHg or DBP (average) ≥90 mmHg. Diabetes was characterized by either self-reported diagnosis, self-reported utilization of medication or insulin to reduce glucose, HbA1c levels of 6.5%, or glucose levels of fasting plasma ≥ 7.0 mmol/L. Hyperlipidemia was characterized by triglycerides at or above 150 mg/dl, total cholesterol at or above 200 mg/dl, LDL at or above 130 mg/dl without using cholesterol-lowering drugs, and HDL lower than 40 mg/dl in men or 50 mg/dl in women.

According to the results of the survey, participants were divided into two groups: nonsmokers and smokers. Smoker status refers to individuals who reported smoking more than 100 cigarettes in their lifetime. The drinking status (yes or no) was determined based on whether alcohol consumption exceeded 12 drinks in the past year.

### 2.4 Statistical analysis

Considering the potential linear or nonlinear association between 25(OH)D serum levels and various mortality outcomes (including all-cause mortality, cardiovascular disease fatality, malignant neoplasm mortality, and other-cause mortality), subjects were categorized into four groups based on quartiles of 25(OH)D serum levels: Q1 (<49.50 nmol/L), Q2 (49.5-67.50 nmol/L), Q3 (67.5-86.7 nmol/L), and Q4 (>86.7 nmol/L).

Continuous variables are presented as mean (standard error, SE), while categorical data is expressed as frequencies (percentages). Kaplan-Meier curves were used to illustrate survival rates based on 25(OH)D serum levels. To visualize the nonlinear correlation between 25(OH)D serum levels and mortality, restricted cubic splines were employed. Cox proportional hazard models were utilized to calculate adjusted hazard ratios (HRs) and their corresponding 95% confidence intervals for different mortality rates associated with 25(OH)D serum levels. Model 1 represents unadjusted results, while Model 2 adjusts for age, sex, race, and PIR. Model 3, which includes all covariates from Model 2, additionally adjusts for education, marital status, BMI, drinking, smoking, diabetes, and hypertension. Finally, a subgroup analysis was utilized to investigate the robust relationship between 25(OH)D serum levels and fatality rate across different populations.

Every result produced from the data was calculated utilizing a weighted data analysis that was founded on intricate sampling. All statistical analyses were performed using R version 4.0.4 (R-project.org), incorporating weighted data analysis based on complex sampling. Statistical significance was indicated by a P-value below 0.05 in all studies.

## 3 Results

### 3.1 Baseline characteristics and demographic information of participants

Based on the final cohort study, a total of 9,271 participants were included, with males representing 43.28% of the cohort. The average age of participants was 69.58 years, and the mean follow-up period was 88.37 months. **Table 1** provides detailed information on survival and mortality rates among the subjects in this study. Age, gender, race, PIR, education level, marital status, BMI, smoking, diabetes, hypertension, and 25(OH)D serum levels were found to have statistically significant associations with mortality rates (P < 0.05). However, no correlation was observed between drinking status and mortality rates. **Table 2** examines the relationship between the aforementioned variables and the independent variable, 25(OH)D serum levels. Higher 25(OH)D serum levels were frequently related to increased survival and reduced mortality rates.

**Table 1:** Baseline of participants with different survival status.

	Overall	Alive	Deceased	P-value
Age (years)	69.58 (6.85)	68.20 (6.23)	73.61 (6.99)	<0.001
Gender				<0.001
Male	4427 (43.28)	2911 (42.08)	1516 (46.82)	
Female	4844 (56.72)	3538 (57.93)	1306 (53.18)	
Race				<0.001
Mexican American	1278 (3.75)	982 (4.15)	296 (2.58)	
Other Hispanic	810 (3.47)	666 (3.68)	144 (2.85)	
Non-Hispanic White	4979 (80.72)	3092 (79.32)	1887 (84.83)	
Non-Hispanic Black	1634 (7.39)	1210 (7.39)	424 (7.36)	
Other Race	570 (4.68)	499 (5.45)	71 (2.39)	
PIR	3.04 (1.55)	3.20 (1.55)	2.56 (1.46)	<0.001
Education				<0.001
Less than high school	1543 (8.21)	980 (6.48)	563 (13.28)	
High school or equivalent	3553 (36.90)	2341 (34.50)	1212 (44.00)	
College or above	4175 (54.88)	3128 (59.03)	1047(42.72)	
Marital status				<0.001
Married	5223 (61.59)	3790 (64.53)	1433 (52.95)	
Separated	3423 (32.75)	2195 (29.62)	1228 (41.94)	
Never married	625 (5.66)	306 (5.85)	116 (5.11)	
BMI (kg/m^2^)	29.32 (6.11)	29.47 (6.02)	28.85 (6.34)	<0.001
Drinking				0.208
No	3609 (36.04)	2565 (35.50)	1044 (37.63)	
Yes	5662 (63.96)	3884 (64.50)	1778 (62.37)	
Smoking				<0.001
No	4452 (48.22)	3291 (50.50)	1161 (41.54)	
Yes	4819 (51.78)	3158 (49.51)	1661 (58.46)	
Diabetes				<0.001
No	6383 (73.98)	4543 (76.14)	1840 (67.64)	
Yes	2888 (26.02)	1906 (23.86)	982 (32.36)	
Hypertension				<0.001
No	2530 (30.53)	1919 (33.45)	611 (21.98)	
Yes	6741 (69.47)	4530 (66.55)	2211 (78.02)	
Serum 25(OH)D	76.73 (30.24)	79.72 (30.42)	67.97 (27.92)	<0.001

All continuous variables shown in **Table 1** were examined for differences using weighted t-tests and expressed using mean ± standard error, and categorical variables were examined using weighted chi-square tests and also expressed as n (%). BMI, body mass index; PIR, poverty income rate.

**Table 2:** Baseline of participants with different serum 25(OH)D levels.

Serum 25(OH)D	Overall	Q1	Q2	Q3	Q4	P-value
N	9271	2320	2317	2322	2312	
Age (years)	69.58 (6.85)	68.88 (6.94)	69.65 (6.92)	69.24 (6.75)	70.20 (6.77)	<0.001
Sex						<0.001
Male	4427 (43.28)	1066 (39.15)	1247 (50.83)	1204 (50.46)	910 (34.55)	
Female	4844 (56.72)	1254 (60.85)	1070 (49.17)	1118 (49.54)	1402 (65.45)	
Race						<0.001
Mexican American	1278 (3.75)	490 (6.97)	380 (4.92)	256 (3.01)	152 (1.80)	
Other Hispanic	810 (3.47)	199 (4.98)	228 (4.16)	229 (3.55)	154 (2.10)	
Non-Hispanic White	4979 (80.72)	802 (65.19)	1190 (78.25)	1445 (84.81)	1542 (87.57)	
Non-Hispanic Black	1634 (7.39)	713 (17.27)	369 (7.38)	261 (4.43)	291 (4.38)	
Other Race	570 (4.68)	116 (5.59)	150 (5.29)	131 (4.20)	173 (4.15)	
PIR	3.04(1.55)	2.61 (1.55)	2.91 (1.56)	3.11 (1.53)	3.31 (1.51)	<0.001
Education						<0.001
Less than high school	1543 (8.21)	483 (11.96)	473 (11.17)	376 (7.94)	211 (4.37)	
High school or equivalent	3553 (36.90)	981 (42.55)	865 (38.01)	885 (37.64)	822 (32.45)	
College or above	4175 (54.88)	856 (45.50)	979 (50.82)	1061 (54.41)	1279 (63.18)	
Marital status						<0.001
Married	5223 (61.59)	1169 (52.26)	1328 (61.90)	1405 (65.08)	1321 (63.64)	
Separated	3423 (32.75)	969(40.43)	840(32.79)	768 (29.49)	846 (31.19)	
Never married	625 (5.66)	182 (7.32)	149 (5.31)	149 (5.43)	145 (5.17)	
BMI (kg/m^2^)	29.315 (6.11)	30.74 (6.69)	29.864 (6.20)	29.105 (5.78)	28.328 (5.78)	<0.001
Drinking						0.002
No	3609 (36.04)	971 (41.42)	856 (33.86)	836 (33.92)	1366 (36.29)	
Yes	5662 (63.96)	1349 (58.58)	1461 (66.14)	1486 (66.08)	1681 (63.71)	
Diabetes						<0.001
No	6383 (73.98)	1434 (66.28)	1600 (72.14)	1668 (75.85)	1681 (77.92)	
Yes	2888 (26.02)	886 (33.72)	717 (27.86)	654 (24.15)	631 (22.08)	
Hypertension						0.027
No	2530 (30.53)	584 (27.86)	658 (29.55)	686 (33.59)	602 (30.14)	
Yes	6741 (69.47)	1736 (72.14)	1659 (70.45)	1636 (66.41)	1710 (69.86)	
Smoking						<0.001
No	4452 (48.22)	1059 (43.85)	1109 (46.13)	1099 (47.20)	1185 (52.88)	
Yes	4819 (51.78)	1261 (56.15)	1208 (53.87)	1223 (52.80)	1127 (47.12)	
Mortality						<0.001
Alive	6449 (74.59)	1450 (63.78)	1554 (69.17)	1617 (75.01)	1828 (83.85)	
Deceased	2822 (25.42)	870 (36.22)	763 (30.84)	705 (25.00)	484 (16.15)	

All continuous variables shown in **Table 1** were examined for differences using weighted one-way ANOVA and expressed using mean ± standard error, and categorical variables were examined using weighted chi-square tests and also expressed as n (%). BMI, body mass index; PIR, poverty income rate.

### 3.2 The relationship between mortality and 25(OH)D serum levels

**Figure 2:**
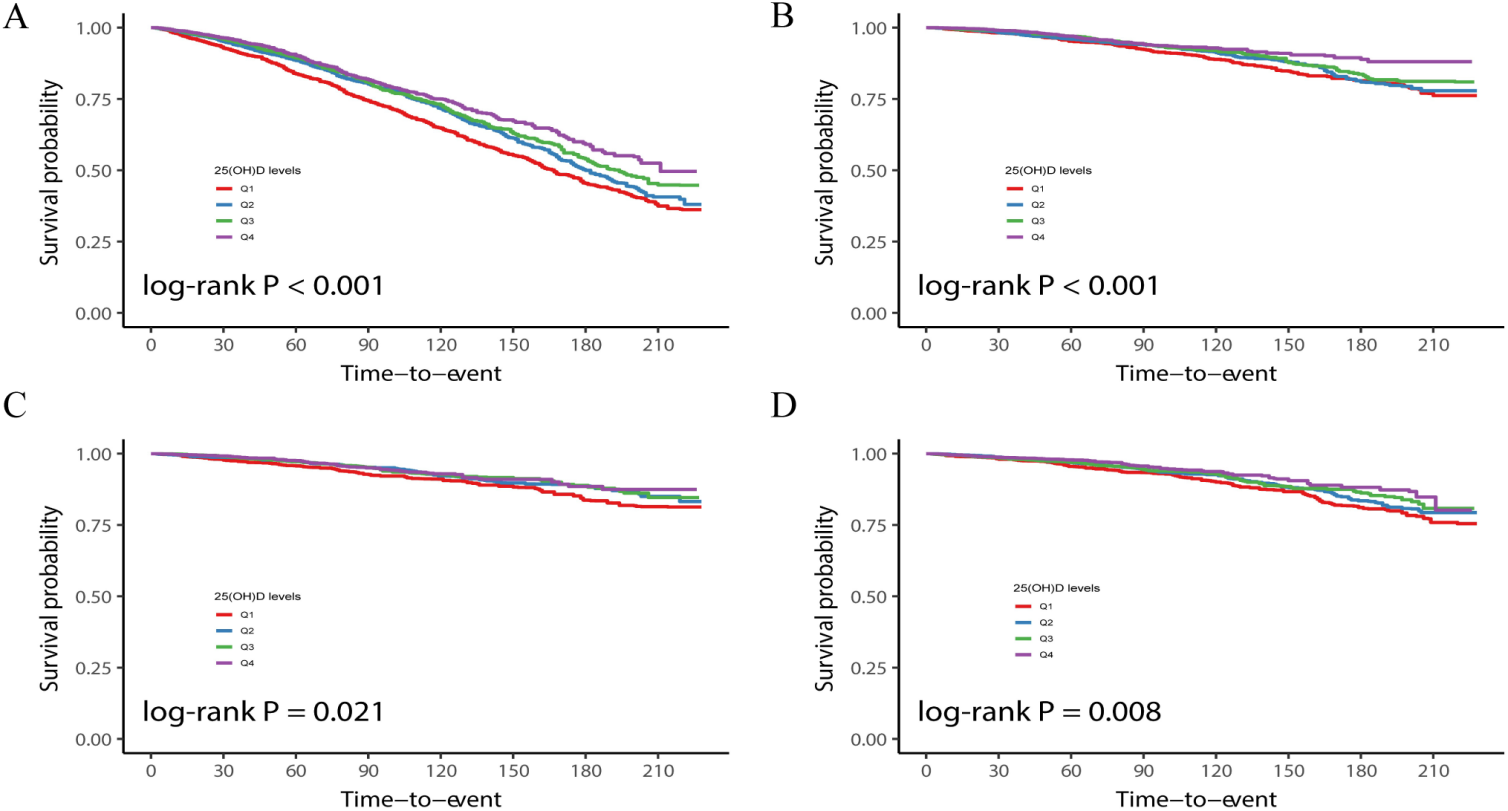
Kaplan-Meier curves showing the relationship between serum 25(OH)D levels quartiles and all-cause **(A)**, CVD **(B)**, malignant neoplasms **(C)**, and other-cause **(D)** mortality.

The cumulative Kaplan-Meier curve of the quartiles of 25(OH)D levels is presented in **Figure 2**. Regarding the overall mortality rate, the cumulative survival probability was significantly lower in the lowest 25(OH)D levels group (P < 0.001). This pattern was consistent for CVD mortality, malignant neoplasm mortality, and other causes of mortality (P < 0.001).

To further investigate the impact of serum 25(OH)D levels on mortality, three Cox regression models were constructed. These models accounted for various factors, including age, sex, ethnicity, education attainment, poverty income rate (PIR), marital status, BMI, smoking status, drinking habits, and history of diabetes or hypertension (Model 3). The adjusted hazard ratios (HRs) and 95% confidence intervals (CIs) were calculated as follows: 0.996 (0.994, 0.998), 0.995 (0.991, 0.999), 0.997 (0.993, 1.002), and 0.994 (0.989, 0.999) for all-cause mortality, CVD mortality, malignant neoplasm mortality, and other-cause mortality, respectively.

The HRs and 95% CIs in **Table 3** calculated for the quartiles of 25(OH)D serum levels, ranging from the lowest to highest categories, were as follows: 1.000 (reference), 0.776 (0.679, 0.887), 0.784 (0.682, 0.901), and 0.697 (0.590, 0.822) for all-cause mortality (P trend <0.001); 1.00 (reference), 0.796 (0.624, 1.015), 0.776 (0.602, 1.001), and 0.673 (0.508, 0.892) for CVD mortality (P trend = 0.007); 1.00 (reference), 0.699 (0.527, 0.927), 0.714 (0.538, 0.947), and 0.712 (0.520, 0.975) for malignant neoplasm mortality (P trend = 0.028); and 1.00 (reference), 0.788 (0.605, 1.028), 0.825 (0.621, 1.097), and 0.649 (0.456, 0.926) for other-cause mortality (P trend = 0.058).

**Table 3:** Cox regression analysis between serum 25(OH)D levels and all-cause and cause-specific mortality.

	Model 1		Model 2		Model 3	
	HR (95%CI)	P	HR (95%CI)	P	HR (95%CI)	P
All-cause mortality						
serum 25(OH)D levels	0.995(0.993,0.998)	<0.001	0.995(0.992,0.997)	<0.001	0.996(0.994,0.998)	<0.001
Q1	ref					
Q2	0.828(0.730,0.939)	0.003	0.721(0.637,0.816)	<0.001	0.776(0.679,0.887)	<0.001
Q3	0.760 (0.654,0.882)	<0.001	0.708(0.616,0.814)	<0.001	0.784(0.682,0.901)	<0.001
Q4	0.675 (0.570,0.800)	<0.001	0.635(0.541,0.745)	<0.001	0.697(0.590,0.822)	<0.001
P for trend	<0.001		<0.001		<0.001	
CVD mortality						
serum 25(OH)D levels	0.994(0.990,0.997)	<0.001	0.993(0.989,0.997)	<0.001	0.995(0.991,0.999)	0.009
Q1						
Q2	0.877(0.713,1.079)	0.215	0.732(0.581,0.922)	0.008	0.796(0.624,1.015)	0.066
Q3	0.768(0.607,0.972)	0.028	0.688(0.536,0.882)	0.003	0.776(0.602,1.001)	0.050
Q4	0.646(0.489,0.853)	0.002	0.599(0.452,0.794)	<0.001	0.673(0.508,0.892)	0.006
P for trend	<0.001		<0.001		0.007	
Malignant neoplasms mortality						
serum 25(OH)D levels	0.996(0.992,1.001)	0.089	0.996(0.991,1.000)	0.073	0.997(0.993,1.002)	0.221
Q1	ref					
Q2	0.752(0.575,0.984)	0.038	0.646(0.491,0.851)	0.002	0.699(0.527,0.927)	0.013
Q3	0.724(0.543,0.966)	0.028	0.645(0.485,0.859)	0.003	0.714(0.538,0.947)	0.019
Q4	0.692(0.507,0.945)	0.021	0.644(0.473,0.876)	0.005	0.712(0.520,0.975)	0.034
P for trend	0.008		0.010		0.028	
Other-cause mortality						
serum 25(OH)D levels	0.993(0.989, 0.998)	0.008	0.993(0.989, 0.998)	0.008	0.994(0.989, 0.999)	0.017
Q1	ref					
Q2	0.827(0.632, 1.080)	0.163	0.745(0.577,0.963)	0.025	0.788(0.605,1.028)	0.079
Q3	0.781(0.591,1.033)	0.084	0.767(0.585,1.005)	0.054	0.825(0.621,1.097)	0.185
Q4	0.634(0.450,0.895)	0.009	0.619(0.442,0.868)	0.005	0.649(0.456,0.926)	0.017
P for trend	0.023		0.011		0.058	

Model 1: no adjusted;

Model 2: adjusted for age, sex, race, and PIR;

Model III: model 2 plus education, marital status, BMI, drinking, smoking, diabetes, and hypertension;

CVD mortality, cardiovascular disease mortality; HR, Hazard Ratio; 95%CI, 95% Confidence Interval.

### 3.3 Dose-response correlation between mortality and 25(OH)D serum levels

**Figure 3:**
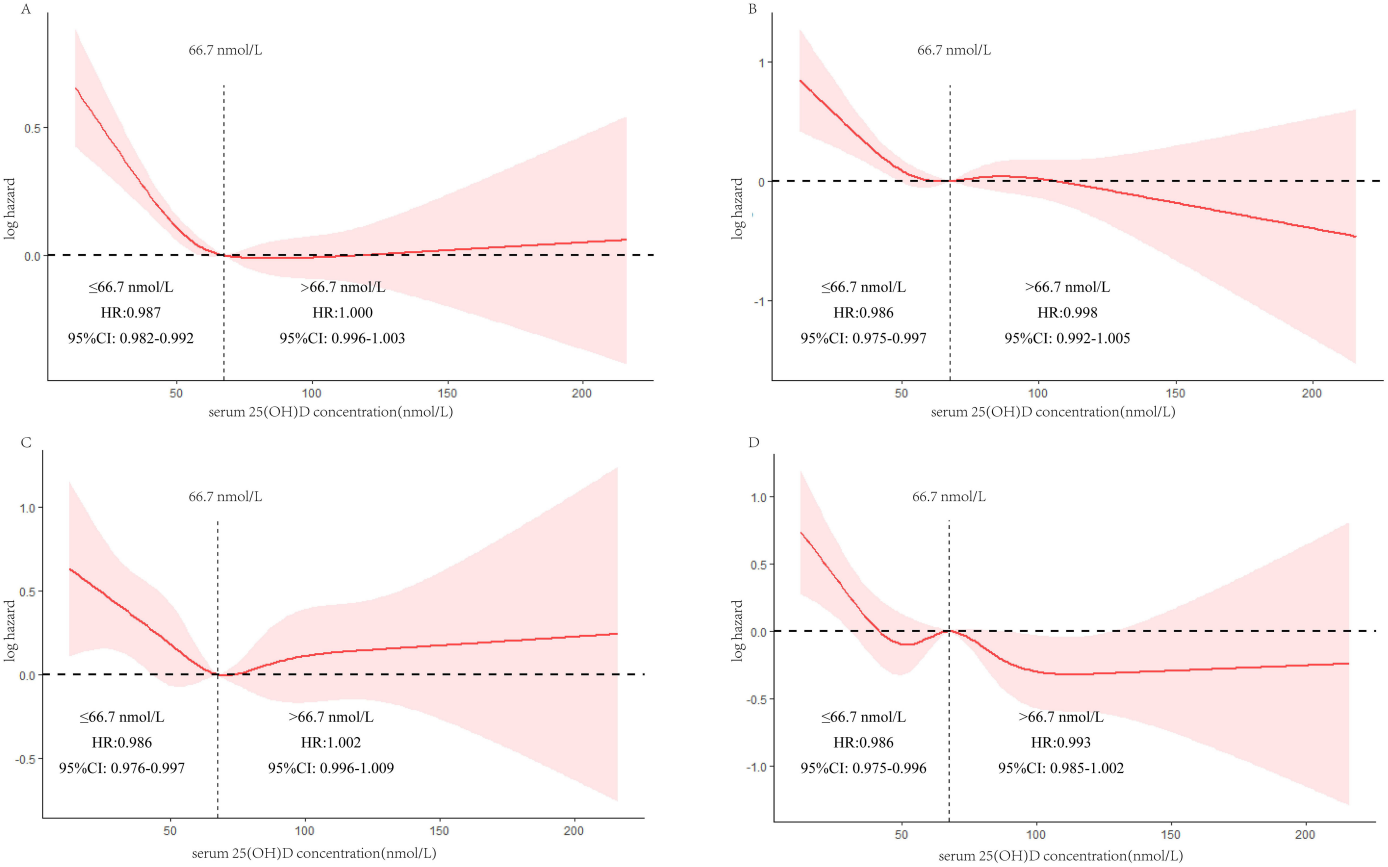
Restricted cubic spline showing the relationship between serum 25(OH)D levels and all-cause **(A)**, CVD **(B)**, malignant neoplasms **(C)**, and other-cause **(D)** mortality.

**Figure 3** illustrates the depiction of the association between mortality and 25(OH)D serum levels using a restricted cubic spline. For all-cause mortality, a notable decrease in mortality was observed initially, followed by a stabilization effect as 25(OH)D serum levels gradually increased. The nonlinear relationship demonstrated a saturation point at approximately 67.5 nmol/L. This nonlinear pattern was similarly observed for CVD mortality, malignant neoplasm mortality, and other-cause mortality, with the saturation point consistently around 67.5 nmol/L. **Table 4** provides further evidence supporting the nonlinear correlation between 25(OH)D serum levels and mortality.

**Table 4:** Saturation effect between serum 25(OH)D concentration and all-cause and cause-specific mortality.

	Saturation value (nmol/L)	≤Saturation value		> Saturation value	
		HR (95%CI)	P-value	HR (95%CI)	P-value
All-cause mortality	67.5	0.987(0.982,0.992)	<0.001	1.000(0.996,1.003)	0.812
CVD mortality	67.5	0.986(0.975,0.997)	0.013	0.998(0.992,1.005)	0.584
Malignant neoplasms mortality	67.5	0.986(0.976,0.997)	0.009	1.002(0.996,1.009)	0.481
Other cause mortality	67.5	0.986(0.975,0.996)	0.006	0.993(0.985,1.002)	0.122

All HRs and their 95%CI were calculated by adjusting for age, sex, race, PIR, education, marital status, BMI, drinking, smoking, diabetes, and hypertension.

### 3.4 Subgroup analysis of the correlation between mortality and 25(OH)D levels

Finally, to investigate the correlation between mortality and 25(OH)D serum levels in greater detail, subgroup analyses were performed on elderly individuals with hyperlipidemia, considering various characteristics. Notably, the robustness of the negative correlation between all-cause mortality and 25(OH)D serum levels was evident across different subgroups, including age, sex, alcohol consumption, hypertension, and diabetes. Interestingly, among non-obese (BMI < 30 kg/m^2^) and smoking elderly individuals with hyperlipidemia, this negative correlation was particularly significant (**Table 5**). Furthermore, robust associations were observed between 25(OH)D serum levels and mortality due to CVD or malignant neoplasms across different subgroups, including age, gender, BMI, tobacco use, alcohol consumption, high blood pressure, and diabetes (**Supplementary Tables S1**, **S2**). In the smoking population, a more pronounced negative relationship was found between other-cause mortality and 25(OH)D serum levels (**Supplementary Table S3**).

**Table 5:** Subgroup analysis of the associations between serum 25 (OH)D levels and all-cause mortality among elderly with hyperlipidemia.

	Serum 25 (OH)D levels (nmol/L)				P for interaction
	Q1	Q2 HR (95%CI)	Q3 HR (95%CI)	Q4 HR (95%CI)	
Age (years)					0.438
≤70 (*n*=4688)	ref	0.641 (0.507,0.810)	0.692 (0.529,0.906)	0.640 (0.457,0.897)	
>70 (*n*=4583)	ref	0.836 (0.731,0.957)	0.793 (0.676,0.929)	0.697 (0.581,0.836)	
Sex					0.978
Male (*n*=4427)	ref	0.736 (0.612,0.885)	0.779 (0.649,0.936)	0.668 (0.537,0.831)	
Female (*n*=4844)	ref	0.782 (0.651,0.940)	0.723 (0.587, 0.890)	0.651 (0.519,0.818)	
BMI (kg/m^2^)					0.017
<30 (*n*=5709)	ref	0.731 (0.608,0.880)	0.689 (0.563,0.842)	0.564 (0.447,0.711)	
≥30 (*n*=3562)	ref	0.760 (0.623,0.928)	0.841 (0.672,1.054)	0.852 (0.664,1.094)	
Smoking					0.018
No (*n*=4452)	ref	0.908 (0.731,1.127)	0.855 (0.684,1.068)	0.789 (0.617,1.009)	
Yes (*n*=4819)	ref	0.670 (0.571,0.786)	0.695 (0.583,0.828)	0.579 (0.468,0.717)	
Drinking					0.164
No (*n*=3609)	ref	0.822 (0.667,1.013)	0.756 (0.612,0.935)	0.742 (0.591,0.931)	
Yes (*n*=5662)	ref	0.702 (0.601,0.818)	0.729 (0.611,0.870)	0.598 (0.480,0.747)	
Diabetes					0.543
No (*n*=6383)	ref	0.827 (0.703,0.973)	0.808 (0.683,0.956)	0.637 (0.509,0.798)	
Yes (*n*=2888)	ref	0.651 (0.540,0.785)	0.658 (0.519,0.834)	0.757 (0.605,0.946)	
Hypertension					0.620
No (n=2530)	ref	0.806 (0.632,1.027)	0.801 (0.611,1.049)	0.687 (0.506,0.933)	
Yes (n=6741)	ref	0.749 (0.646,0.868)	0.743 (0.631,0.876)	0.660 (0.551,0.789)	

1. Wald test was performed to examine the interaction between continuous serum 25 (OH)D concentration and stratification variables.

2. Cox proportional hazard models were used to estimate the HRs (95% CIs) by adjusting for all covariates.

## 4 Discussion

In this nationally representative cohort study, an L-shaped relationship was observed between the risk of 25(OH)D serum and the risk of cardiovascular disease (CVD), malignant neoplasms, all-cause mortality, and other-cause mortality. Specifically, an increase in 25(OH)D serum levels was associated with a decreased risk of mortality from various causes, including CVD, malignant neoplasms, and other causes. These relationships were independent of conventional risk factors such as age, lifestyle factors, BMI, diabetes, and hypertension. We visualized the correlation between 25(OH)D serum levels and different causes of mortality using restricted cubic splines and determined the optimal levels of 25(OH)D to reduce mortality rates through threshold analysis. Subgroup analysis further confirmed the consistent negative relationship between 25(OH)D serum levels and mortality. This study is the initial investigation that focuses on the correlation between 25(OH)D serum levels and cause-specific or all-cause mortality within the hyperlipidemic geriatric population.

To date, numerous studies have already established a close association between 25(OH)D serum levels and overall mortality in populations with diverse characteristics. Large-scale studies conducted on American elderly individuals have independently demonstrated a negative correlation between 25(OH)D serum levels and CVD and all-cause mortality (15). Similar findings were subsequently found in another study of a large national cohort from different regions (16–19). Subsequently, growing attention has been given to the association between 25(OH)D serum levels and mortality in populations with various characteristics. Notably, negative associations between 25(OH)D serum levels and mortality have been reported in individuals with diabetes, osteoarthritis patients, those in the prediabetic population, patients with fatty liver and metabolic dysfunction, rectal cancer patients, and individuals with severe diseases (20–27). However, studies focusing on hyperlipidemic populations remain limited.

Hyperlipidemia is a prevalent condition in modern society, which is considered the most critical risk factor for CVD, making it the primary contributor to adult mortality in the United States (28). Multiple studies have identified the associations between hyperlipidemia and CVD, including induction of oxidative stress, inflammatory cardiac fibrosis, reduced autophagy, decreased microvascular density, and altered mitochondrial function in cardiomyocytes (29, 30), which provide insights into the mechanisms underlying hyperlipidemia induced increase of CVD risk. Consequently, our study mainly focused on the elderly population with hyperlipidemia, who are known to be particularly susceptible to CVD. A retrospective research study found that increasing serum 25(OH) D concentration may help treat hyperlipidemia in vitamin D deficiency (31). In this study, a close relationship was discovered between increased levels of serum 25(OH)D and decreased CVD mortality among elderly individuals with hyperlipidemia. This effect may be attributed to the anti-inflammatory, antioxidative, and enhanced mitochondrial respiration properties of 25(OH)D (32). Apart from its well-established association with CVD, hyperlipidemia has also been linked to an elevated risk of various types of cancer (33–36), which indicates that hyperlipidemia is a risk factor for tumor development. In this study, the correlation between elevated 25 (OH)D serum levels and reduced mortality from tumors has also been discovered.

While numerous public health studies have established an association between vitamin D deficiency and increased risks of all-cause mortality and cause-specific mortality, few have guided the appropriate levels of 25(OH)D to mitigate these risks. The Endocrine Society has outlined a reliable clinical practice guideline categorizing vitamin D levels into four groups: severely deficient (<25.00 nmol/L), moderately inadequate (25.00-49.99 nmol/L), insufficient (50.00-74.99 nmol/L), and adequate (>75.00 nmol/L) (37). However, the optimal level remains controversial for several reasons. Lack of standardization of testing is the cause of this problem, and measures should be taken to overcome it. It is important to note that this classification focuses on individuals at risk of vitamin D insufficiency and does not recommend routine screening for deficiency in low-risk individuals. Considering the potential adverse effects of vitamin D deficiency on bone and overall health, especially when serum 25(OH)D levels are less than 30 nmol/L (<12 ng/mL), it may be useful to prioritize screening of at-risk populations (9). In two large cohort studies involving individuals with diabetes and prediabetes, approximately 80% of the population exhibited vitamin D deficiency. Similarly, within a Chinese cohort study of 1,834 elderly adults, only 3.7% of participants met the Endocrine Society’s recommended threshold for adequate 25(OH)D serum levels (>75 nmol/L) (38). Moreover, several studies have suggested that the current thresholds for 25(OH)D sufficiency may be too stringent for certain populations, highlighting the need for more appropriate guidelines to inform population health. In this study, participant 25(OH)D serum levels were divided into quartiles to explore their linear or nonlinear relationships with cause-specific and all-cause mortality. A nonlinear relationship was observed, and an optimal 25(OH)D level of 67.5 nmol/L was identified, providing valuable insights into the role of 25(OH)D serum levels in reducing mortality rates.

In the present study, we observed a stronger negative correlation between serum 25(OH)D levels and all-cause mortality in older adults with hyperlipidemia who had a BMI less than 30. This may be due to the fact that lower BMI indicative of better overall health and lifestyle is associated with better vitamin D status as lower body fat percentage may increase vitamin D bioavailability (39). Thus, the protective effect of vitamin D may be more pronounced in individuals with lower BMI. In addition, we found a more significant negative correlation between serum 25(OH)D levels and all-cause mortality in the smoking hyperlipidemic elderly population. This observation may be related to the increased oxidative stress and inflammation induced by smoking, biological processes that can be mitigated by the anti-inflammatory and antioxidant properties of vitamin D (40, 41). Furthermore, since smoking is a strong predictor of cardiovascular disease and vitamin D has been shown to have positive effects on cardiovascular health, including lowering blood pressure and improving endothelial function (42), this may be more pronounced in smokers.

### 4.1 Strengths and limitations

Our study was based on NHANES 2001-2016 data covering 9,271 hyperlipidemic elderly participants, which provided us with sufficient statistical power to detect the association between serum 25(OH)D levels and mortality. In addition, the long follow-up period of our study (mean 88.37 months) allowed us to observe more stable long-term results, increasing the reliability of our findings. Although the reported findings yield intriguing conclusions, it is important to acknowledge certain limitations within this research. Firstly, as an observational study, establishing a causal relationship is not feasible. Secondly, 25(OH)D serum levels were assessed based on a single measurement, failing to capture participants’ status consistently throughout the follow-up period. Thirdly, despite meticulous adjustment for numerous confounding factors, the presence of unmeasured or unknown residual confounders cannot be entirely ruled out. Fourthly, the investigation did not delve into the specific effects of distinct vitamin D supplements on the association between 25(OH)D levels and mortality rates. Therefore, further research is imperative to elucidate the potential role of vitamin D supplementation in this domain. Finally, it is noteworthy that African-American and Hispanic adults are more susceptible to 25(OH)D insufficiency (37), but the subgroup analysis did not account for the potential impact of race on the observed association.

## 5 Conclusion

Within a cohort study comprising elderly individuals with hyperlipidemia, there exists a significant independent association between higher serum levels of 25(OH)D and a decreased risk of cause-specific mortality as well as all-cause mortality.

## Data Availability

The original contributions presented in the study are included in the article/**Supplementary Material**. Further inquiries can be directed to the corresponding author.
