# Supplementary material for: Saturation association between serum 25-hydroxyvitamin D levels and mortality in elderly people with hyperlipidemia: a population-based study from the NHANES (2001-2016)
Source: Front Endocrinol (Lausanne). 2024 Oct 2;15:1382419. doi: 10.3389/fendo.2024.1382419 (PMC11479873; doi:10.3389/fendo.2024.1382419)
Supplement: Supplementary file 1 [file Table1.docx]

| **Table S1.** Stratified analysis of the associations between serum 25(OH)D concentration and CVD mortality among elderly with hyperlipidemia. | | | | | |
| --- | --- | --- | --- | --- | --- |
|  | Serum 25(OH) concentrations (nmol/L) | | | |  |
| Characteristic | Q1 | Q2 | Q3 | Q4 | P for interaction |
| Age (years) |  |  |  |  | 0.158 |
| ≤70(n = 4688) | ref | 0.512 (0.318, 0.824) | 0.603 (0.359, 1.013) | 0.480 (0.244, 0.946) |  |
| >70 (n = 4583) | ref | 0.947  (0.737, 1.216) | 0.844 (0.659, 1.082) | 0.738 (0.542, 1.005) |  |
| Sex |  |  |  |  | 0.441 |
| Male  (n = 4427) | ref | 0.735 (0.522, 1.034) | 0.786 (0.540, 1.145) | 0.712 (0.479, 1.058) |  |
| Female  (n = 4844) | ref | 0.868 (0.651, 1.157) | 0.721 (0.498, 1.043) | 0.595 (0.398, 0.890) |  |
| BMI (kg/m^2^) |  |  |  |  | 0.377 |
| <30 (n = 5709) | ref | 0.775 (0.553, 1.085) | 0.760 (0.547, 1.055) | 0.560 (0.378, 0.830) |  |
| ≥30 (n = 3562) | ref | 0.771 (0.520, 1.143) | 0.747 (0.475, 1.174) | 0.828 (0.485, 1.415) |  |
| Smoking |  |  |  |  | 0.207 |
| No (n = 4452) | ref | 1.047 (0.714, 1.535) | 0.960 (0.658, 1.402) | 0.736 (0.460, 1.177) |  |
| Yes (n = 4819) | ref | 0.623 (0.448, 0.866) | 0.626 (0.441, 0.889) | 0.569 (0.393, 0.824) |  |
| Drinking |  |  |  |  | 0.596 |
| No (n = 3609) | ref | 0.821 (0.575, 1.171) | 0.625 (0.436, 0.897) | 0.660 (0.427, 1.018) |  |
| Yes (n = 5662) | ref | 0.751 (0.552, 1.023) | 0.848 (0.593, 1.212) | 0.639 (0.441, 0.927) |  |
| Diabetes |  |  |  |  | 0.457 |
| No (n = 6383) | ref | 0.781 (0.597, 1.021) | 0.768 (0.547, 1.077) | 0.579 (0.340, 0.837) |  |
| Yes (n = 2888) | ref | 0.840 (0.543, 1.299) | 0.754 (0.485, 1.170) | 0.812 (0.517, 1.277) |  |
| Hypertension |  |  |  |  | 0.354 |
| No (n = 2530) | ref | 0.606 (0.351, 1.046) | 0.815 (0.499, 1.330) | 0.482 (0.260, 0.893) |  |
| Yes (n = 6741) | ref | 0.850 (0.663, 1.090) | 0.769 (0.575, 1.029) | 0.701 (0.507, 0.971) |  |

1.Wald test was performed to examine the interaction between continuous serum 25(OH)D concentration and stratification variables.

2.Cox proportional hazards models were used to estimate the HRs (95% CIs) by adjusting for all covariates.
