# Supplementary material for: Saturation association between serum 25-hydroxyvitamin D levels and mortality in elderly people with hyperlipidemia: a population-based study from the NHANES (2001-2016)
Source: Front Endocrinol (Lausanne). 2024 Oct 2;15:1382419. doi: 10.3389/fendo.2024.1382419 (PMC11479873; doi:10.3389/fendo.2024.1382419)
Supplement: Supplementary file 2 [file Table2.docx]

| **Table S2.** Stratified analysis of the associations between serum 25(OH)D concentration and malignant neoplasms mortality among elderly with hyperlipidemia. | | | | | | | |
| --- | --- | --- | --- | --- | --- | --- | --- |
|  |  |  | Serum 25(OH) concentrations (nmol/L) | | | |  |
| Characteristic | | | Q1 | Q2 | Q3 | Q4 | P for interaction |
| Age (years) | | |  |  |  |  | 0.921 |
| ≤70 (n = 4688) | | | ref | 0.682 (0.413, 1.125) | 0.551 (0.342, 0.886) | 0.628 (0.350, 1.126) |  |
| >70 (n = 4583) | | | ref | 0.690 (0.495, 0.960) | 0.751 (0.535, 1.055) | 0.715 (0.505, 1.013) |  |
| Sex | | |  |  |  |  | 0.674 |
| Male (n = 4427) | | | ref | 0.638 (0.413, 0.985) | 0.700 (0.466, 1.052) | 0.598 (0.350, 1.022) |  |
| Female (n = 4844) | | | ref | 0.712 (0.500, 1.012) | 0.628 (0.396, 0.994) | 0.715 (0.481, 1.062) |  |
| BMI (kg/m^2^) | | |  |  |  |  | 0.191 |
| <30 (n = 5709) | | | ref | 0.631 (0.438, 0.909) | 0.577 (0.411, 0.810) | 0.556 (0.411, 0.810) |  |
| ≥30 (n = 3562) | | | ref | 0.704 (0.470, 1.054) | 0.839 (0.537, 1.313) | 0.870 (0.499, 1.517) |  |
| Smoking | | |  |  |  |  | 0.779 |
| No (n = 4452) | | | ref | 0.660 (0.438, 0.995) | 0.604 (0.378, 0.963) | 0.675 (0.405, 1.126) |  |
| Yes (n = 4819) | | | ref | 0.666 (0.480, 0.923) | 0.694 (0.500, 0.965) | 0.650 (0.449, 0.941) |  |
| Drinking | | |  |  |  |  | 0.379 |
| No (n = 3609) | | | ref | 0.557 (0.362, 0.857) | 0.540 (0.336, 0.870) | 0.649 (0.399, 1.055) |  |
| Yes (n = 5662) | | | ref | 0.729 (0.521, 1.022) | 0.732 (0.509, 1.054) | 0.672 (0.448, 1.010) |  |
| Diabetes | | |  |  |  |  | 0.884 |
| No (n = 6383) | | | ref | 0.782 (0.571, 1.070) | 0.739  (0.547, 0.998) | 0.670 (0.465, 0.963) |  |
| Yes (n = 2888) | | | ref | 0.437 (0.250, 0.763) | 0.517 (0.290, 0.924) | 0.718 (0.385, 1.338) |  |
| Hypertension | | |  |  |  |  | 0.192 |
| No (n = 2530) | | | ref | 0.891 (0.557, 1.426) | 0.931 (0.579, 1.497) | 1.021 (0.573, 1.820) |  |
| Yes (n = 6741) | | | ref | 0.606 (0.440, 0.833) | 0.601 (0.430, 0.841) | 0.588 (0.419, 0.825) |  |
