# Supplementary material for: Saturation association between serum 25-hydroxyvitamin D levels and mortality in elderly people with hyperlipidemia: a population-based study from the NHANES (2001-2016)
Source: Front Endocrinol (Lausanne). 2024 Oct 2;15:1382419. doi: 10.3389/fendo.2024.1382419 (PMC11479873; doi:10.3389/fendo.2024.1382419)
Supplement: Supplementary file 3 [file Table3.docx]

| **Table S3.** Subgroup analysis of the associations between serum 25(OH) concentration and other-cause mortality among elderly with hyperlipidemia. | | | | | |  |
| --- | --- | --- | --- | --- | --- | --- |
|  | Serum 25(OH) concentrations (nmol/L) | | | |  | |
| Characteristic | Q1 | Q2 | Q3 | Q4 | P for interaction | |
| Age (years) |  |  |  |  | 0.733 | |
| ≤70 (n = 4688) | 1 | 0.567 (0.338, 0.952) | 0.719 (0.451, 1.148) | 0.572 (0.259, 1.261) |  | |
| >70 (n = 4583) | 1 | 0.915 (0.685, 1.221) | 0.867 (0.634, 1.186) | 0.678 (0.462, 0.993) |  | |
| Sex |  |  |  |  | 0.63 | |
| Male (n = 4427) | 1 | 0.926 (0.704, 1.218) | 0.857 (0.619, 1.188) | 0.614 (0.397, 0.951) |  | |
| Female (n = 4844) | 1 | 0.672 (0.449, 1.007) | 0.772 (0.520, 1.145) | 0.616 (0.392, 0.968) |  | |
| BMI (kg/m^2^) |  |  |  |  | 0.52 | |
| <30 (n = 5709) | 1 | 0.788 (0.554, 1.122) | 0.739 (0.511, 1.070) | 0.579 (0.383, 0.876) |  | |
| ≥30 (n = 3562) | 1 | 0.729 (0.463, 1.148) | 0.872 (0.542, 1.401) | 0.650 (0.372, 1.135) |  | |
| Smoking |  |  |  |  | 0.013 | |
| No (n = 4452) | 1 | 0.949 (0.658, 1.370) | 1.020 (0.677, 1.537) | 0.856 (0.533, 1.374) |  | |
| Yes (n = 4819) | 1 | 0.671 (0.471, 0.956) | 0.690 (0.488, 0.975) | 0.476 (0.311, 0.728) |  | |
| Drinking |  |  |  |  | 0.426 | |
| No (n = 3609) | 1 | 0.822 (0.532, 1.268) | 0.852 (0.531, 1.366) | 0.713 (0.418, 1.217) |  | |
| Yes (n = 5662) | 1 | 0.713 (0.520, 0.976) | 0.729 (0.510, 1.043) | 0.536 (0.331, 0.868) |  | |
| Diabetes |  |  |  |  | 0.958 | |
| No (n = 6383) | 1 | 0.940 (0.642, 1.376) | 0.923 (0.639, 1.334) | 0.640 (0.402, 1.020) |  | |
| Yes (n = 2888) | 1 | 0.532 (0.338, 0.835) | 0.606 (0.372, 0.988) | 0.653 (0.379, 1.125) |  | |
| Hypertension |  |  |  |  | 0.483 | |
| No (n = 2530) | 1 | 1.195 (0.758, 1.884) | 0.966 (0.545, 1.653) | 0.969 (0.565, 1.653) |  | |
| Yes (n = 6741) | 1 | 0.703 (0.514, 0.963) | 0.762 (0.560, 1.036) | 0.561 (0.384, 0.819) |  | |

1.Wald test was performed to examine the interaction between continuous serum 25(OH)D concentration and stratification variables.

2.Cox proportional hazards models were used to estimate the HRs (95% CIs) by adjusting for all covariates.
